# Reorganisation and the Volunteer Medical Officers

**Published:** 1907-06-22

**Authors:** 


### The Royal Army Medical Corps Section.

### REORGANISATION AND THE VOLUNTEER MEDICAL OFFICERS.

An important speech was recently made at the annual dinner of the Scottish Volunteer Medical Officers' Association by Colonel Beatson, C.B., M.D., whose knowledge and experience endow his utterances on this subject with special authority. Col. Beatson insists that the value of outside influence and encouragement to the Volunteers cannot be over-estimated. It has often helped to overcome many difficulties. This fact has a special importance at the moment, from the circumstance that Mr. Haldane's proposals, when adopted, will change the whole status, character, management, and name of the present Volunteer force. The conditions of service in the new force will be entirely at variance with that spontaneous and voluntary enrolment for home defence which has been the proud and distinguishing feature of the existing Volunteer system. In such circumstances Col. Beatson expressed surprise that the contemplated changes have not evoked a stronger expression of public opinion than they have done. The public were under the misapprehension that the new scheme had the considered and deliberate approval of the Volunteer commanding officers. Although certain commanding officers were asked by Mr. Haldane to express an opinion, and to offer suggestions as to the carrying out of a scheme already thought out and decided upon, had the commanding officers been asked to formulate a scheme, it would have been a very different one from that now under the consideration of Parliament. Certainly they would never have consented to sweep away, as the War Office proposed to sweep away, a splendid body of men with an honourable history and cherished associations, such as the Volunteers; nor, having inflicted on them this indignity, would they have been asked to enrol next day under a new designation and under absolutely new conditions of service. With Mr. Haldane's aims no fault can be found, but his means are faulty and savour of ingratitude, for they will ruthlessly sweep away a force that has done splendid service, and has been more sinned against than sinning. Mr. Haldane has fallen into the error of confounding enthusiasm and military spirit.

We propose to devote a section of The Hospital to R.A.M.C. matters, including the new field ambulance for the regular Army, and the Volunteer medical officers, their ambulance, and position in the service. Col. Beatson has well enforced the lesson taught by actual warfare in South Africa. He has shown clearly that the old arrangements for rendering aid to the sick and wounded of an army on service in the field must be changed, and he heartily approves the action of the present director-general of the Army Medical Service by impressing on the military authorities the value to the Army of sanitation. The effect of the splendid organisation of the medical service of the Japanese army in the Russo-Japanese war must be to render the arguments of our own director-general unanswerable. In the Russo-Japanese war, for the first time in the history of warfare, there were fewer deaths from disease than from casualties. In former wars the usual mortality had been four deaths from disease to one from casualties. In the Russo-Japanese war it was four deaths from casualties to one from disease, a result eight hundred per cent. better than the average of history. To prevent disease there must be a screen of sanitary scouts to precede every Army unit, who would test the water of all the rivers, examine all villages for disease, inspect its food supplies, fruit and wells, and certify or condemn them in accordance with their actual condition. This was the Japanese plan, and everything was done by them to protect the troops against disease. The Japanese results are a splendid triumph for preventive medicine, and constitute an unanswerable argument for a well equipped medical department for the British Army, with independent powers. Sir Alfred Keogh may be relied upon to so reorganise our Army medical service as to render it capable of securing identical or even better results, than the Japanese, should the nation, unfortunately, again be involved in war.

Passing on to the arrangements for rendering aid to the sick and wounded of a regular army when on service in the field, it is essential that the old dual arrangement of bearer company and field hospital, which sometimes led to friction, shall be abolished, for the Boers rightly claimed that the Geneva Convention does not provide for the neutrality of a field hospital, and so made captures of our field hospitals and upheld their legal right to do so. In re-organising the medical arrangements for service in the field, the new system recently introduced into the field ambulance for the regular Army aims at the removal of many defects. It does not go quite far enough in certain directions, but it has brought about one great improvement by reorganising the system of field medical units. The field medical unit now combines in one unit the bearer company and field hospital under the new title of the Field Ambulance, a term which is recognised by the Geneva Convention. It is essential to the successful working of any field ambulance of an efficient army medical service that the officers of that service shall have a force of men, equivalent to a strength equal to two men per company, independently borne on the strength, and entitled to the capitation grant, to serve as regimental bearers for the regimental medical officer. The new Army regulations make a change in the source of the regimental aid, but they do not in our judgment adequately cover the point just mentioned. It is good that all the authorised bandsmen of a unit should be trained to act as stretcher-bearers, though this does not apply to the Royal Artillery, but on actual service the bandsmen would be required for other purposes very often. This is a point which we hope Sir Alfred Keogh will drive home with the War Office authorities.

The existing Volunteer medical service has still to be placed upon a satisfactory footing. It ought to consist of one united corps, the R.A.M.C.

(Volunteers). To it every V.M.O. should belong, and under every regimental surgeon there should be a fixed and recognised body of men for sanitary and ambulance duties alone. These men must be supernumerary to the regiment, and a capitation grant must be drawn separately for each of them. Every regiment must have its own ambulance equipment under its own medical officer. That officer should, if he wishes, have the right to wear the uniform of his regiment, but he must also have the corps uniform, and for the latter only should he get an allowance. It is to be hoped that one effect of Mr. Haldane's scheme will be to arouse infinitely greater public interest in the work of the Volunteers, so that municipalities may interest themselves in the question and provide certain funds on the American system, with a view to secure that every Volunteer regiment shall be so housed and equipped that it can be got ready for active service, at any time, on two hours' notice. The National Guards in the United States under such a scheme, so far as their best regiments are concerned, have attained to this state of efficiency. Why should the United Kingdom wait?

